# Simulating ideal assistive devices to reduce the metabolic cost of walking with heavy loads

**DOI:** 10.1371/journal.pone.0180320

**Published:** 2017-07-12

**Authors:** Christopher L. Dembia, Amy Silder, Thomas K. Uchida, Jennifer L. Hicks, Scott L. Delp

**Affiliations:** 1 Department of Mechanical Engineering, Stanford University, Stanford, California, United States of America; 2 Department of Bioengineering, Stanford University, Stanford, California, United States of America; 3 Department of Orthopaedic Surgery, Stanford University, Stanford, California, United States of America; Norwegian University of Science and Technology, NORWAY

## Abstract

Wearable robotic devices can restore and enhance mobility. There is growing interest in designing devices that reduce the metabolic cost of walking; however, designers lack guidelines for which joints to assist and when to provide the assistance. To help address this problem, we used musculoskeletal simulation to predict how hypothetical devices affect muscle activity and metabolic cost when walking with heavy loads. We explored 7 massless devices, each providing unrestricted torque at one degree of freedom in one direction (hip abduction, hip flexion, hip extension, knee flexion, knee extension, ankle plantarflexion, or ankle dorsiflexion). We used the Computed Muscle Control algorithm in OpenSim to find device torque profiles that minimized the sum of squared muscle activations while tracking measured kinematics of loaded walking without assistance. We then examined the metabolic savings provided by each device, the corresponding device torque profiles, and the resulting changes in muscle activity. We found that the hip flexion, knee flexion, and hip abduction devices provided greater metabolic savings than the ankle plantarflexion device. The hip abduction device had the greatest ratio of metabolic savings to peak instantaneous positive device power, suggesting that frontal-plane hip assistance may be an efficient way to reduce metabolic cost. Overall, the device torque profiles generally differed from the corresponding net joint moment generated by muscles without assistance, and occasionally exceeded the net joint moment to reduce muscle activity at other degrees of freedom. Many devices affected the activity of muscles elsewhere in the limb; for example, the hip flexion device affected muscles that span the ankle joint. Our results may help experimentalists decide which joint motions to target when building devices and can provide intuition for how devices may interact with the musculoskeletal system. The simulations are freely available online, allowing others to reproduce and extend our work.

## Introduction

Wearable robotic devices are currently used to help restore mobility to individuals following a stroke, a spinal cord injury, or the loss of a limb [1–4]. Other potential uses for assistive devices are to reduce injury risk for those carrying heavy loads, such as firefighters [5], laborers [6], and soldiers [7]. A common goal of assistive device designers is to reduce the metabolic cost of walking. Yet, reducing the metabolic cost of walking using a device is difficult—despite decades of effort [8–11], this has been accomplished only recently [9,12–19]. Designers are making progress on overcoming the challenges of large subject-to-subject variability in performance [14], minimizing the metabolic penalty of carrying a device [9], and designing effective training protocols [20,21]. One of the largest challenges is understanding the complex neuromusculoskeletal adaptations (short- and long-term) that occur when the body is augmented with assistive devices. For example, even if a device reduces muscle activity, the device may not reduce metabolic cost or muscle fiber power [22,23].

Using experiments, researchers have learned much about providing assistance during gait. For example, metabolic cost can be sensitive to actuation timing [13,24], metabolic cost can be reduced with a strictly passive device [9], and unilateral assistance (on one leg only) can affect the activity of muscles on the unassisted leg [25]. Nevertheless, there is still much to be learned. In particular, discovering the metabolic effect of separately assisting each joint of the leg during walking would be a significant milestone in understanding human–device interaction [26]. Achieving this milestone through experiments alone is currently impractical, as device developers would need to invest substantial time and money into designing and refining a device for each joint [9]. Further, an experimental approach provides limited ability to evaluate how the timing and magnitude of applied torques affect the performance of a device, independent of the device’s mass, its comfort, the subject’s adaptations, and other practical factors.

Musculoskeletal simulations can complement experiments in designing assistive devices. Simulations have revealed the breakdown of energy consumption during walking into stance and swing costs [27], have shown that wearable robots can negatively affect muscle fiber mechanics [22,28], and have suggested that asymmetric gaits are metabolically optimal for unilateral amputees with prostheses [29]. Using simulations to accurately predict the effects of an assistive device is a substantial challenge, as modeling the effects of device mass, device comfort, and training protocols is very difficult. However, a strength of simulation is that, rather than mathematically characterizing all features of a device, we can explore hypothetical ideal devices—devices that are massless, provide lossless transmission of torque to the limb, and have no torque or power limits—and thus compare different types of devices (e.g., hip vs. knee) independent of other practical challenges. We can also optimize the devices for a specific objective, such as minimizing muscle activation. Moreover, we can perform such simulations over many subjects and scenarios [30–34]. When paired with a model of muscle energy consumption [27,32,35,36], simulations can also predict how devices might affect the energy consumed by individual muscles. Our simulations and energy estimates can be used to inform decisions about which joints to target with devices and to estimate how real-world devices affect muscle activity. In turn, the results of experimental studies can be used to further validate and improve the predictive capability of simulations.

### Study objectives

In this study, we examined how ideal (massless with no torque or power limits) assistive devices affect metabolic cost when walking with heavy loads. We used musculoskeletal simulation to evaluate 7 ideal bilateral assistive devices that each provided one joint moment (uniarticular) in one direction (unidirectional): hip abduction, hip flexion, hip extension, knee flexion, knee extension, ankle plantarflexion, or ankle dorsiflexion. The simulations tracked motion capture data of loaded walking. We used an optimization procedure to simultaneously optimize the behavior of the devices and predict changes in muscle activity in response to applied device torques. Specifically, at regular intervals throughout the motion, we solved for the device torques that minimized the sum of squared muscle activations while tracking measured kinematics of loaded walking without assistance. We did not optimize any attributes of the device other than the torque profiles of its two actuators. We repeated these simulations for 7 subjects and multiple gait cycles. This study is methodologically similar to a recent study from our research group that also examined hypothetical assistive devices, but the devices were bidirectional, multi-joint, and assisted running [33].

We had three specific aims. First, we sought to determine which of the 7 devices had the highest ratio of metabolic savings to device power (on average across subjects); we used this ratio as an “efficiency” metric to identify devices that provide relatively large metabolic savings with a small power requirement. Second, we aimed to compare features between the optimal torque profiles of the devices and the corresponding net joint moments produced by muscles during loaded walking. Third, we sought to assess how each device might change lower-limb muscle activity.

Our study presents the first muscle-actuated simulations of heavily loaded walking. We performed the simulations with the open-source OpenSim software package (version 3.3) [37,38], which was also used for much of the simulation work described earlier [22,30,32,33] and contains validated muscle [39] and metabolics [27,32,35] models. The data and code required to reproduce the results are available at https://simtk.org/home/assistloadwalk.

## Methods

### Experiments

We collected motion capture data from 7 male individuals (age 25 ± 5 years, height 1.86 ± 0.04 m, mass 84 ± 15 kg; mean ± standard deviation; S1 Table). Subjects were recruited between May and September of 2013 by word of mouth from the Stanford University campus and the surrounding communities. Data were collected for four conditions:

1. without load at a freely selected speed (1.46 ± 0.15 m/s; referred to as the *no load* condition),
2. without load at approximately 80% of the speed from the subject’s *no load* trials (1.20 ± 0.10 m/s),
3. while carrying 38 kg on the torso at a freely selected speed (1.27 ± 0.09 m/s; *loaded*), and
4. while carrying 38 kg on the torso at approximately the same speed as in the subject’s *no load* trials (1.48 ± 0.09 m/s).

All subjects performed these conditions in the order listed above, and all subjects who enrolled in the study completed all conditions.

For each condition, the subjects completed at least 3 overground trials and a single 7-minute treadmill trial (no incline). During the overground trials, we measured optical marker trajectories, ground reaction forces and moments, and muscle activity. The trajectories of 41 markers were collected at 100 Hz with an 8-camera optical motion capture system (Motion Analysis Corp., Santa Rosa, CA, USA). Ground reaction forces and moments were collected at 2000 Hz from 3 floor-mounted force plates (Bertec Corp., Columbus, OH, USA). Synchronously, we collected the activity of 10 lower-limb muscles at 2000 Hz using surface electromyography (EMG) sensors (Trigno™; Delsys Inc., Boston, MA, USA). Each overground trial captured approximately one gait cycle. We used the treadmill trial (Woodway Pro XL; Woodway Inc., Waukesha, WI, USA) to estimate whole-body metabolic energy consumption using indirect calorimetry (Quark b^2^; COSMED, Rome, Italy). We analyzed the final minute of data to estimate metabolic rate [40], using the percent change over the last 3 minutes to verify that steady-state was reached.

The added mass in the loaded conditions was split between a backpack (8 kg, including the backpack itself) and 3 weight vests containing lead (30 kg, including the vests; Hyperware, Austin, TX, USA). The backpack did not have a hip belt but was worn tightly, and the weight in each weight vest was evenly distributed between the front and back of the vest.

Only the *loaded* condition was used to study assistive devices. The *no load* and *loaded* conditions were used to validate the metabolics model. The remaining two conditions were used only for normalizing EMG sensor readings; because subjects did not perform maximum voluntary contractions, the fourth condition listed above—our most intensive—typically provided the highest measured muscle activity.

The Stanford University Institutional Review Board approved our experimental protocol and all subjects provided written informed consent.

### Simulations of experiments

We generated simulations of the *no load* and *loaded* conditions. We refer to the simulations of the *loaded* condition as *no assistance* because the *no assistance* simulations are the baseline for the *assisted* simulations (described in the “Simulations of assisted loaded walking” section, below).

We used a three-dimensional musculoskeletal model that is based on 21 cadavers and 24 young healthy humans [41]. The model contains 39 degrees of freedom, though we locked 8 of them that we deemed nonessential for our study (bilateral ankle eversion, toe flexion, wrist flexion, and wrist deviation). For our simulations of the *loaded* condition, we modeled the load as a hollow cylindrical channel (height 40 cm, inner radius 13 cm, outer radius 15 cm) with uniform density, welded to the torso.

Our simulation workflow began with scaling the geometry of the generic musculoskeletal model to match the anthropometry of each of our subjects, using the OpenSim Scale Tool. Additionally, we scaled the maximum isometric forces of the muscles according to a regression equation based on subject mass and height [42]. For each subject and condition, we simulated 3 of the overground trials. For each of these trials, we generated joint angle trajectories using OpenSim’s Inverse Kinematics (IK) Tool. We assigned greater tracking weights to anatomical markers than to tracking markers, the latter of which were attached to marker plates on the thigh and shank.

We used OpenSim’s Residual Reduction Algorithm (RRA) Tool to reduce the residual forces (applied to the pelvis) resulting from inconsistencies between force plate data, marker data, and the musculoskeletal model [37]. We ran RRA twice for each trial: first, to generate an adjusted model (*RRA-model*), and then to generate adjusted kinematics (*RRA-kinematics*). We combined all adjusted models from each run of *RRA-model* for the same subject and condition (by averaging the suggested mass adjustments) to create a single adjusted model for each subject and condition. This strategy helps to avoid overfitting the model to the experimental data from any particular trial, which may occur when using a separate adjusted model for each trial. For the *loaded* condition, we used *RRA-model* to adjust the mass and location of the load. We then produced adjusted kinematics for each trial by running *RRA-kinematics*, using the single adjusted model and the kinematics from IK as input. Finally, we generated muscle-driven simulations of the overground trials with OpenSim’s Computed Muscle Control (CMC) Tool [43], using the single adjusted model and the adjusted kinematics.

#### Objective function in Computed Muscle Control

CMC solves for muscle excitations that can produce the observed walking motion while minimizing the sum of squared muscle activations at regular intervals in the motion. Specifically, CMC’s objective function, *J*, consists of an effort term, *J*_effort_, and a term associated with modeling and measurement error, *J*_error_:

$$J=J_{\text{effort}}+J_{\text{error}},\#$$ (1)

$$J_{\text{effort}}=\underset{i\in M}{\sum }a_{i}^{2},\#$$ (2)

$$J_{\text{error}}=\underset{i\in R}{\sum }{\left(\frac{f_{i}}{w_{f,i}}\right)}^{2}.\#$$ (3)

The effort term (Eq 2) depends only on the activation *a* of the set of muscles *M* in the model. The error term (Eq 3) penalizes the force or moment *f* applied by the set of *reserve* and *residual* actuators *R* in the model. Reserve actuators apply small joint moments to compensate for unmodeled passive structures (e.g., ligaments) and potential muscle weakness, and residual actuators apply the residual forces explained above. The weighting factor *w*_*f*_ is adjusted to make the reserves and residuals much more costly to use compared to the muscles; in OpenSim, this factor is the actuators’ “optimal force” property.

We generated 21 *no load* simulations and 21 *no assistance* (*loaded*) simulations (7 subjects, 3 trials per condition).

### Simulations of assisted loaded walking

#### Assistive devices

We also used the CMC Tool to design and predict the effect of 7 hypothetical assistive devices. The new simulations built upon the *no assistance* simulations described above. Each device was uniarticular (acted at a single degree of freedom), unidirectional (acted in only one direction), and added bilaterally (to both legs). We considered 6 sagittal-plane devices: hip flexion, hip extension, knee flexion, knee extension, ankle plantarflexion, and ankle dorsiflexion. We also considered a hip abduction device because a preliminary analysis showed that carrying a load can substantially increase the activity of the hip abductors [44]. We chose unidirectional devices because they are common in the literature and provide a clear picture of how the devices affect muscle activity.

We were interested in the maximum possible benefit that each of these devices could provide. As such, we modeled the devices as massless, with lossless transmission of force to the limb, and as not having any limits on the torques or mechanical power they provide. The devices were implemented in OpenSim as CoordinateActuators, using control bounds to define their unidirectionality. Each device was bilateral and thus consisted of two CoordinateActuators, one on each leg. The two actuators were controlled independently.

#### Objective function in Computed Muscle Control

In the *assisted* simulations, the CMC algorithm controlled both the muscles and the device. As a result, the objective function included the torques τ applied by the two actuators (left and right legs) of the device:

$$J_{\text{effort}}=\underset{i\in M}{\sum }a_{i}^{2}+{\left(\frac{\tau_{\text{left}}}{w_{\tau ,\text{left}}}\right)}^{2}+{\left(\frac{\tau_{\text{right}}}{w_{\tau ,\text{right}}}\right)}^{2}.\#$$ (4)

To maximize the use of the device in place of muscles, we set the weighting factors *w*_τ_ to a large value (1000 N-m) so that using the device had a negligible penalty. The CMC optimization played the two roles of finding the optimal device behavior and predicting changes in muscle activity. The *assisted* simulations tracked the same kinematics (and used the same ground reaction forces) as the *no assistance* simulations on which they were based, so the net joint moments throughout the motion were conserved for all degrees of freedom. With the aid of the device to achieve those same net joint moments, overall muscle coordination could change to arrive at a lower *J*_effort_.

From each of the 21 *no assistance* simulations, we generated 7 simulations of assistance (one per device), giving a total of 147 simulations of assistance.

### Validation of simulations

Joint angles and net joint moments (S1 Fig) for the *no load* and *loaded* simulations were qualitatively similar to those from other studies of loaded walking [45,46]. The primary exception to this was the hip flexion moment reported by Huang and Kuo [46], which we might expect to be different because the load they used was different from the one we used: in their study, the entire load was in a backpack, which had a hip belt.

The timing of muscle activity (S2 Fig) was similar between simulations and EMG measurements for the gluteus maximus, gluteus medius, vastus lateralis, vastus medialis, gastrocnemius, soleus, and tibialis anterior. The discrepancy in timing for the medial hamstrings, biceps femoris, and gastrocnemius resulted from excessive passive knee force. The magnitude of muscle activity was similar between simulations and EMG for all but a few recorded muscles. Large tibialis anterior activity during swing resulted from excessive soleus force (passive force in dorsiflexion, and lingering activity during deactivation; in our model, the soleus can exert much larger ankle moments than the tibialis anterior). The simulated anterior gluteus medius activity was greater than EMG likely because the gluteus medius is a fan-shaped muscle and it is difficult to experimentally measure the activity of the entire muscle. The simulated vasti activity was lower than EMG because EMG was normalized by the greatest activity we observed across conditions and it is unlikely the vasti were maximally activated in any of our conditions. The implications of these discrepancies on our results are mentioned in the Discussion.

The next three subsections present a set of error metrics we computed based on suggestions by Hicks et al. (page 20 in [47]). These metrics were computed over all simulations we performed.

#### Kinematics errors

The simulations tracked lower-limb joint angles with a root-mean-square (RMS) error of 0.3 degrees, averaged across lower-limb degrees of freedom and simulations; the maximum error was 2.2 degrees. The RMS error in marker trajectories between CMC simulations and experimental data had a mean value of 2.1 cm across lower-limb markers and simulations; the maximum error across time, lower-limb markers, and simulations was 9.9 cm. Excluding the two distal toe markers, the maximum error was 6.9 cm; we expected larger errors for the distal toe markers (mostly in swing) because we locked the ankle eversion degree of freedom. We believe these marker errors are sufficiently small, given that this study’s conclusions are largely qualitative.

#### Residual errors

The RMS magnitudes of the residual force and moment had mean values of 12 N and 19 N-m, respectively, across simulations. The maximum magnitudes of the residual force and moment over time and simulations were 49 N and 59 N-m, respectively. Expressed as a percentage of the peak ground reaction force (GRF) magnitude, the RMS residual force magnitude had a mean value of 0.9% across simulations; the peak residual force magnitude had a maximum value of 4.2%. These percentages are within the guideline of 5% provided by Hicks et al. [47]. The residual moments, however, were greater than the guideline of 1% suggested by Hicks et al.: expressed as a percentage of the product of average COM height and peak GRF magnitude, the RMS residual moment had a mean value of 1.4%; the peak residual moment magnitude had a maximum value of 4.8%. Despite exceeding the residual moment guideline, we do not expect the magnitudes of our residual moments to affect the main conclusions of our study: having accurate net joint moments for the muscle-actuated degrees of freedom is more important for generating realistic muscle behavior, and our net joint moments compared favorably with those from the literature [45,46].

#### Reserve errors

The RMS error between generated (from muscles and the device) and tracked net joint moments (i.e., the *reserve* moment in OpenSim terminology) had a mean value of 0.14 N-m across degrees of freedom and simulations; the maximum error across time, degrees of freedom, and simulations was 21 N-m. The ratio of the RMS error to the maximum absolute tracked net joint moment had a mean value of 0.2% over degrees of freedom and simulations; this meets the guideline of 5% provided by Hicks et al. [47] and indicates that muscles and devices supplied nearly all the required net joint moments. The ratio of the peak error to the maximum absolute tracked joint moment had a maximum value of 19%; large peaks of this magnitude occurred for only two trials of a single subject, and spanned less than 10% of the gait cycle (providing limited opportunity to affect metabolic cost estimates), and therefore do not affect the study’s conclusions.

### Metabolics model

**Fig 1:**
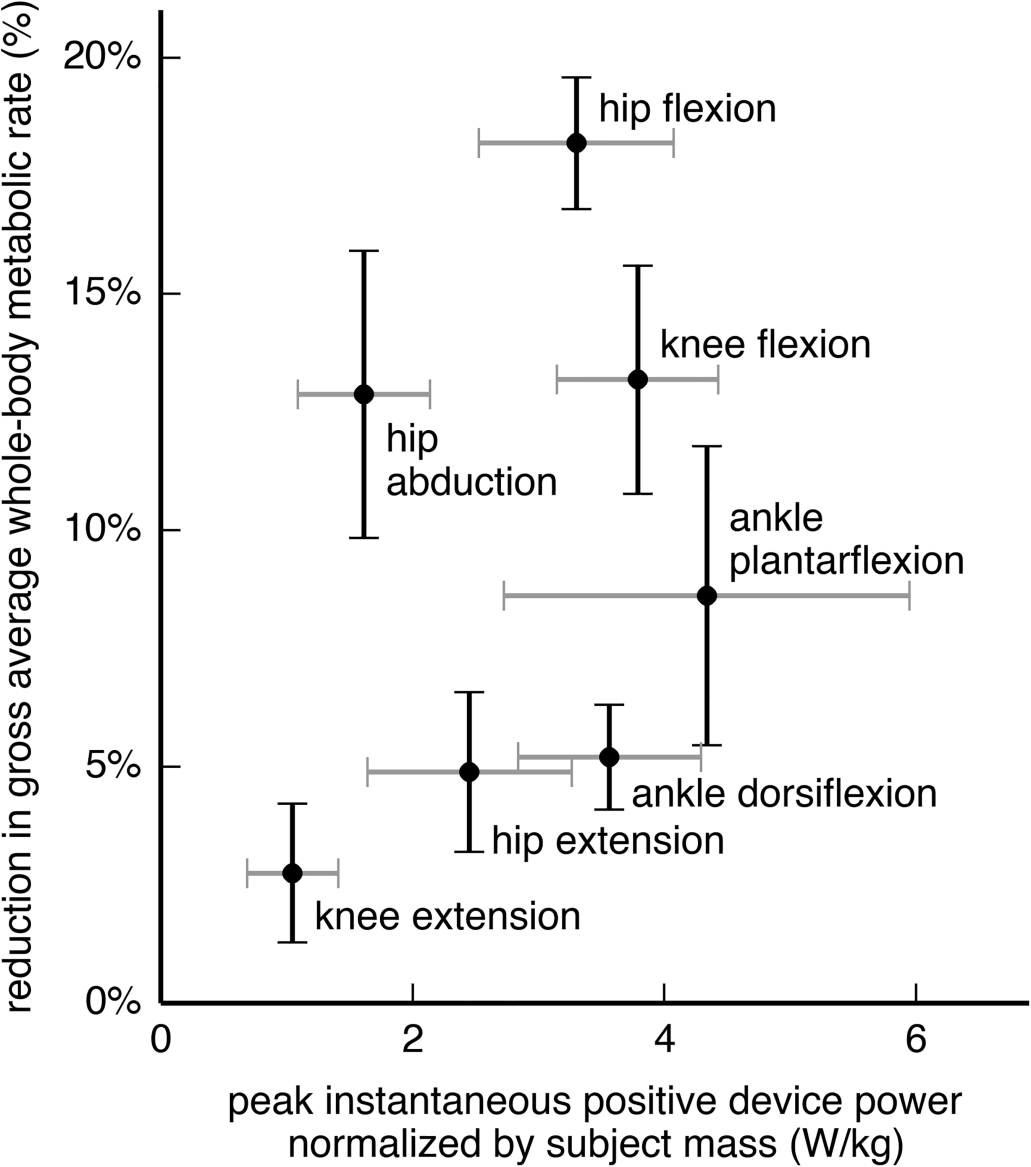
Reduction in metabolic rate from each device, compared to its peak positive power. The vertical axis shows the percent reduction in gross whole-body metabolic rate, averaged over the gait cycle. The horizontal axis shows the peak instantaneous positive power of the device over the gait cycle, normalized by subject mass. The peak positive device power is the maximum, over time, of the sum of the positive power output of both actuators (left and right legs). Each crosshair provides the mean and standard deviation across 7 subjects for a single device. The hip flexion, knee flexion, and hip abduction devices provided greater metabolic savings than the ankle plantarflexion device (Tukey post-hoc test, p < 0.05).

To estimate metabolic energy consumption from the simulations, we used a metabolics model developed by Umberger et al. [27,35] with some modifications [32]. To employ this metabolics model, we used the Umberger2010MuscleMetabolicsProbe in OpenSim 3.3. To compute *gross average whole*-*body metabolic rate* (Fig 1), we used the following procedure: we summed the rate of energy consumption of all muscles, added a basal rate (1.2 W/kg [35]), then integrated the resulting whole-body rate over the gait cycle and divided by the duration of the gait cycle.

The preferred speed and stride length of some subjects caused us to have insufficient force plate data to simulate a complete gait cycle for several trials (16 *no load* trials and 6 *loaded* trials, affecting 29% of the simulations of assistance). To estimate average whole-body metabolic rate for simulations derived from these trials, we averaged the instantaneous whole-body rate over half a gait cycle (exploiting the approximate mediolateral symmetry of walking). To assess the effect of this approximation on our results, we also computed an average rate for 5 equally spaced half-gait cycles throughout the available data. We found negligible difference in our results between using the mean over those 5 average rates and using a single half-gait cycle.

#### Validation of the metabolics model

We validated the metabolics model by comparing its estimates of the gross average whole-body metabolic rate for the *no load* and *loaded* conditions to the respective estimates from indirect calorimetry (S3 Fig). We normalized both estimates by subject mass and walking speed; because the treadmill speed could only be set in increments of 0.1 mph (0.045 m/s), subjects walked at slightly different speeds between the overground and treadmill trials. This normalization yielded a quantity known as *metabolic cost of transport* [48], which describes the energy required to travel a unit distance (1 W/kg/(m/s) = 1 J/m/kg). The simulations estimated the normalized gross average metabolic rate for the *loaded* condition to be 5.77 W/kg/(m/s); indirect calorimetry estimated this quantity to be 5.83 W/kg/(m/s). The relative error in normalized gross average whole-body metabolic rate between simulation and indirect calorimetry had a mean value of 11% across subjects and both conditions. In this study, we were primarily interested in predicting the percent change in metabolic rate between conditions and across subjects. The simulations estimated a 40% increase in metabolic rate from the *no load* to the *loaded* condition, on average across subjects; indirect calorimetry estimated a 48% increase. This discrepancy suggests the simulations also underestimated the change in metabolic rate with assistance.

### Evaluating the devices

To evaluate the benefit of a device, we computed the percent reduction in gross average whole-body metabolic rate for each subject and trial (21 data points for each device). We then computed the mean and standard deviation of this percent reduction over subjects. To evaluate the cost of carrying a device, we obtained the peak positive power performed by the device (sum of the positive power from the two actuators), then normalized the peak power by subject mass, and finally computed the mean and standard deviation over subjects (resulting in units of W/kg). We used peak power as a surrogate for the mass of the device: if one assumes a fixed specific power for the actuators, peak device power can be used to estimate the actuators’ mass [14,49]. Although this estimate ignores the mass of non-actuator components, energy regeneration, and passive assistance strategies, it allows us to begin addressing a key limitation of our study (the massless nature of the devices). We also computed the ratio between the absolute reduction in metabolic rate (normalized by subject mass; units of W/kg) and the peak device power; this unitless “efficiency” metric attempts to capture the preference for devices that provide large metabolic savings with a small power requirement.

In addition, we computed the average positive and average negative device power (using the sum of power output from the two actuators, normalized by subject mass); average positive device power can be used to estimate the battery life of untethered devices. Lastly, we computed the ratio between the absolute reduction in metabolic rate and average positive device power. This (unitless) ratio provided a metric similar to the “performance index” from Sawicki and Ferris [50] and the “muscle–tendon efficiency” from Mooney et al. [14].

#### Statistical testing

To compare the devices for the metrics listed above, we employed a linear mixed model (fixed effect: device; random effect: subject) with analysis of variance (ANOVA) tests and Tukey post-hoc pairwise tests [51]. We used a significance level of α = 0.05. The data for the statistical analyses consisted of 49 observations (7 subjects and 7 devices); we averaged over the 3 trials for each subject–device pair to remove hierarchical structure from our data [52]. The statistical tests were performed with R [53–55].

### Metabolic rate attributed to joint motions

To understand how the devices affected metabolic cost, we estimated the metabolic cost of actuating individual *joint motions*. We define a joint motion as one direction of a degree of freedom (e.g., hip flexion and hip extension are two joint motions). Uchida et al. [32] presented a method for partitioning the metabolic rate of biarticular muscles in the sagittal plane; here, we generalize that method to muscles that actuate more than two degrees of freedom. We first partitioned the instantaneous metabolic rate of each muscle *i*, *Ė*_*i*_(*t*), across the joint motions *g* that the muscle actuated in proportion to its moment arms *r*_*i*,*g*_(*t*) ≥ 0 about those joint motions:

$${\dot{E}}_{i,g}\left(t\right)=\frac{r_{i,g}\left(t\right)}{\sum_{k\in G}r_{i,k}\left(t\right)}{\dot{E}}_{i}\left(t\right),\#$$ (5)

where *Ė*_*i*,*g*_(*t*) is the metabolic rate of muscle *i* attributed to joint motion *g*, and *G* is the set of joint motions in the model. We obtained the instantaneous metabolic rate for a joint motion, *Ė*_*g*_(*t*), by summing the contributions from all muscles *M*:

$${\dot{E}}_{g}\left(t\right)=\underset{i\in M}{\sum }{\dot{E}}_{i,g}\left(t\right).\#$$ (6)

One can recover the whole-body metabolic rate by summing *Ė*_*g*_(*t*) across all joint motions (and adding the basal rate), though we report results for only a subset of joint motions. The set of joint motions *G* did not include those from constrained degrees of freedom such as ankle eversion.

We summed *Ė*_*g*_(*t*) over the same joint motion for both legs, then averaged this sum over the gait cycle (using only half a gait cycle when necessary; see the “Metabolics model” section, above), and normalized this average rate by the subject’s mass and walking speed. We then found the mean and standard deviation of the normalized average joint motion metabolic rate over subjects, for the *no assistance* simulations and for each device.

Other methods for apportioning metabolic cost to joint motions, such as using the product of moment arm and angular velocity [33], may be equally valid. We drew qualitative conclusions from this analysis that we believe would hold under different apportioning methods.

## Results

### Device performance

All 7 of our ideal devices significantly decreased average whole-body metabolic rate from that of walking without assistance (Fig 1, vertical axis; p < 0.05). The hip flexion (18.2% reduction), knee flexion (13.2%), and hip abduction (12.9%) devices provided greater savings than the other devices tested, including the ankle plantarflexion device (8.6%; Tukey post-hoc test, p < 0.05). The remaining 3 devices had smaller effects on metabolic rate—the knee extension (2.8%), hip extension (4.9%), and ankle dorsiflexion (5.2%) devices.

The peak instantaneous positive power (Fig 1, horizontal axis; Table 1, column c) for the ankle plantarflexion device (4.34 W/kg) was significantly greater than that for all other devices except the knee flexion device (Tukey post-hoc test, p < 0.05). The hip abduction device had the greatest ratio of metabolic savings to peak positive device power (0.63; Table 1, column f) and the greatest ratio of metabolic savings to average positive device power (2.37; Table 1, column g).

**Table 1:** Device performance and power.

device	reduction in gross average whole-body metabolic rate		device power (W/kg)			ratio of reduction in metabolic rate (b) to positive device power	
	(a) relative (%)	(b) absolute (W/kg)	(c) peak positive	(d) average positive	(e) average negative	(f) peak (b)/(c)	(g) average (b)/(d)
hip abduction	12.9 ± 3.0	0.93 ± 0.18	1.61 ± 0.52	0.42 ± 0.11	−0.27 ± 0.09	0.63 ± 0.14	2.37 ± 0.58
hip flexion	18.2 ± 1.4	1.33 ± 0.14	3.30 ± 0.77	1.04 ± 0.14	−0.29 ± 0.13	0.42 ± 0.09	1.29 ± 0.12
hip extension	4.9 ± 1.7	0.36 ± 0.14	2.45 ± 0.81	0.57 ± 0.18	−0.00 ± 0.00	0.15 ± 0.05	0.62 ± 0.10
knee flexion	13.2 ± 2.4	0.96 ± 0.18	3.79 ± 0.64	1.10 ± 0.25	−0.37 ± 0.10	0.26 ± 0.03	0.89 ± 0.10
knee extension	2.8 ± 1.5	0.21 ± 0.13	1.05 ± 0.36	0.17 ± 0.08	−0.17 ± 0.08	0.19 ± 0.07	1.18 ± 0.21
ankle plantarflexion	8.6 ± 3.2	0.65 ± 0.28	4.34 ± 1.61	0.51 ± 0.21	−0.18 ± 0.07	0.15 ± 0.01	1.26 ± 0.08
ankle dorsiflexion	5.2 ± 1.1	0.38 ± 0.10	3.56 ± 0.73	0.60 ± 0.11	−0.25 ± 0.03	0.11 ± 0.02	0.64 ± 0.11

This table shows the (a) relative and (b) absolute reduction in gross average whole-body metabolic rate achieved by each assistive device, and each device’s (c) peak positive, (d) average positive, and (e) average negative power. Device power quantities are evaluated over the sum of the power output of both actuators (left and right legs). Quantities in columns (b)–(e) are normalized by subject mass. Column (f) shows the ratio of the relative reduction in average whole-body metabolic rate to peak positive device power (i.e., column (b) over column (c)); column (g) is similar but uses average positive device power (i.e., column (d)) in the denominator. All columns are reported as mean ± standard deviation across 7 subjects.

By partitioning whole-body metabolic cost into the metabolic cost of actuating individual *joint motions* (S4 Fig), we arrived at two key insights. First, most devices only partially reduced the metabolic rate of its associated joint motion: the reduction was less than half for the hip extension and knee extension devices. Second, many devices affected the metabolic rate attributed to joint motions other than the one actuated by the device; for example, the hip abduction and knee flexion devices both reduced the metabolic rate attributed to hip flexion.

### Optimal device torques and powers

**Fig 2:**
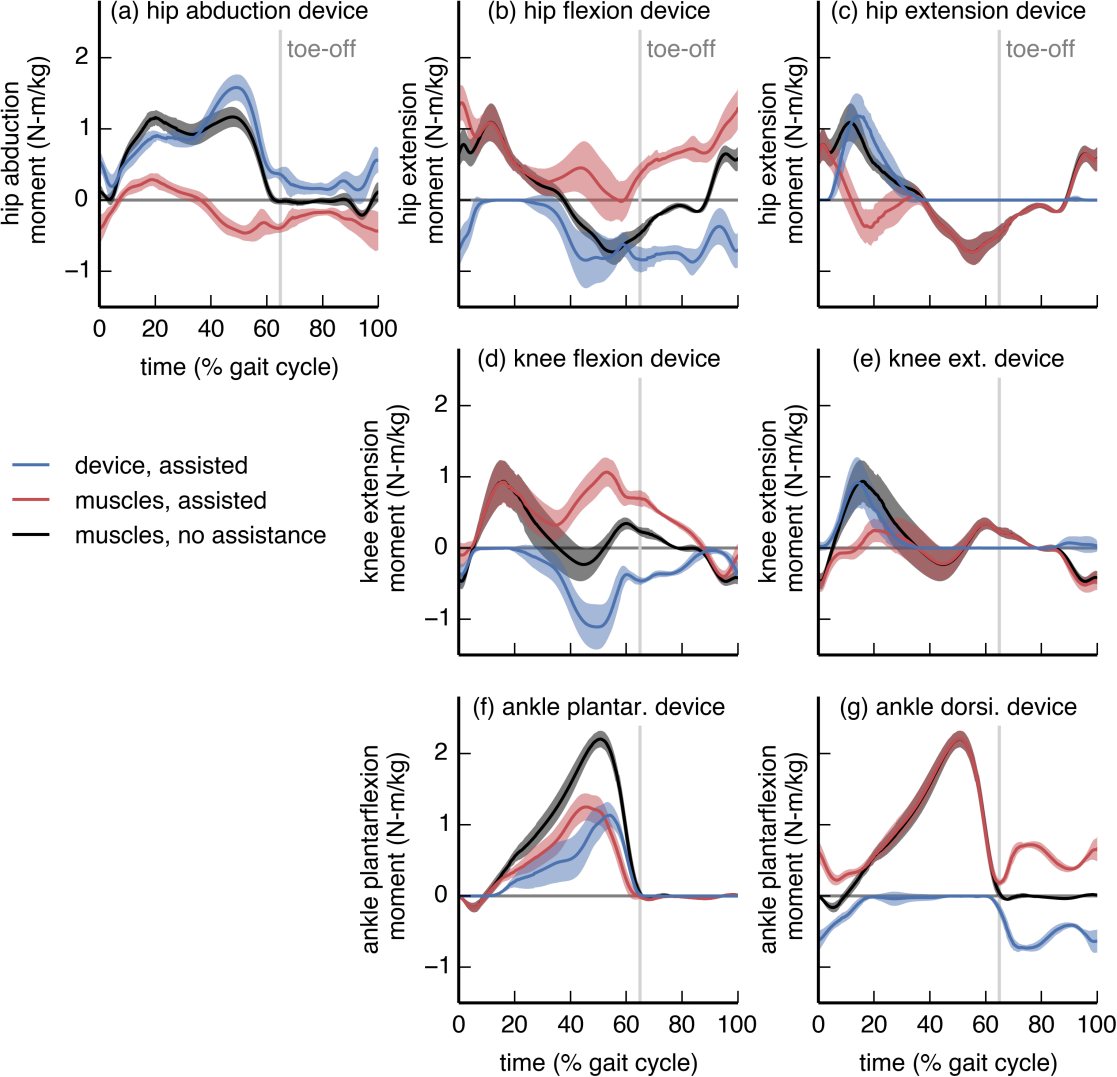
Device torque profiles compared to net joint moments. The device actuator torque (blue), net joint moment generated by muscles in the presence of assistance (red), and net joint moment generated by muscles when not being assisted (black) are shown for each device. The moments are normalized by subject mass. Curves are averages over 7 subjects; shaded regions indicate ±1 standard deviation. The “muscles, no assistance” moment is the tracked net joint moment for both the *no assistance* and the *assisted* simulations; the “muscles, assisted” and “device, assisted” moments approximately sum to the “muscles, no assistance” moment. In many cases, the optimal device torque differed substantially from the net joint moment generated by muscles without assistance.

The optimal torque for most devices differed substantially from the net joint moment of the assisted degree of freedom (Fig 2). In some cases, the device torque exceeded the net joint moment; this was evident for the hip flexion (Fig 2B), knee flexion (Fig 2D), and ankle dorsiflexion (Fig 2G) devices. In these cases, the device torque and net muscle moment opposed each other; the “Muscle-generated joint moments” section, below, explains why this behavior was optimal. The hip flexion and knee flexion devices were active primarily during late stance and throughout swing. The hip extension (Fig 2C) and knee extension (Fig 2E) devices were active primarily during early stance, when the hip and knee required net extension moments. The hip and knee extension devices were not active in late stance or during swing, despite the requirement for net extension moments during these phases. The ankle plantarflexion device (Fig 2F) was active throughout most of stance, and reached its peak moment in late stance, when the plantarflexor muscles generate their largest moment. The peak torque from the ankle plantarflexion device, averaged across subjects (“subject-average”), was 52% of the peak subject-average net ankle plantarflexion moment.

**Fig 3:**
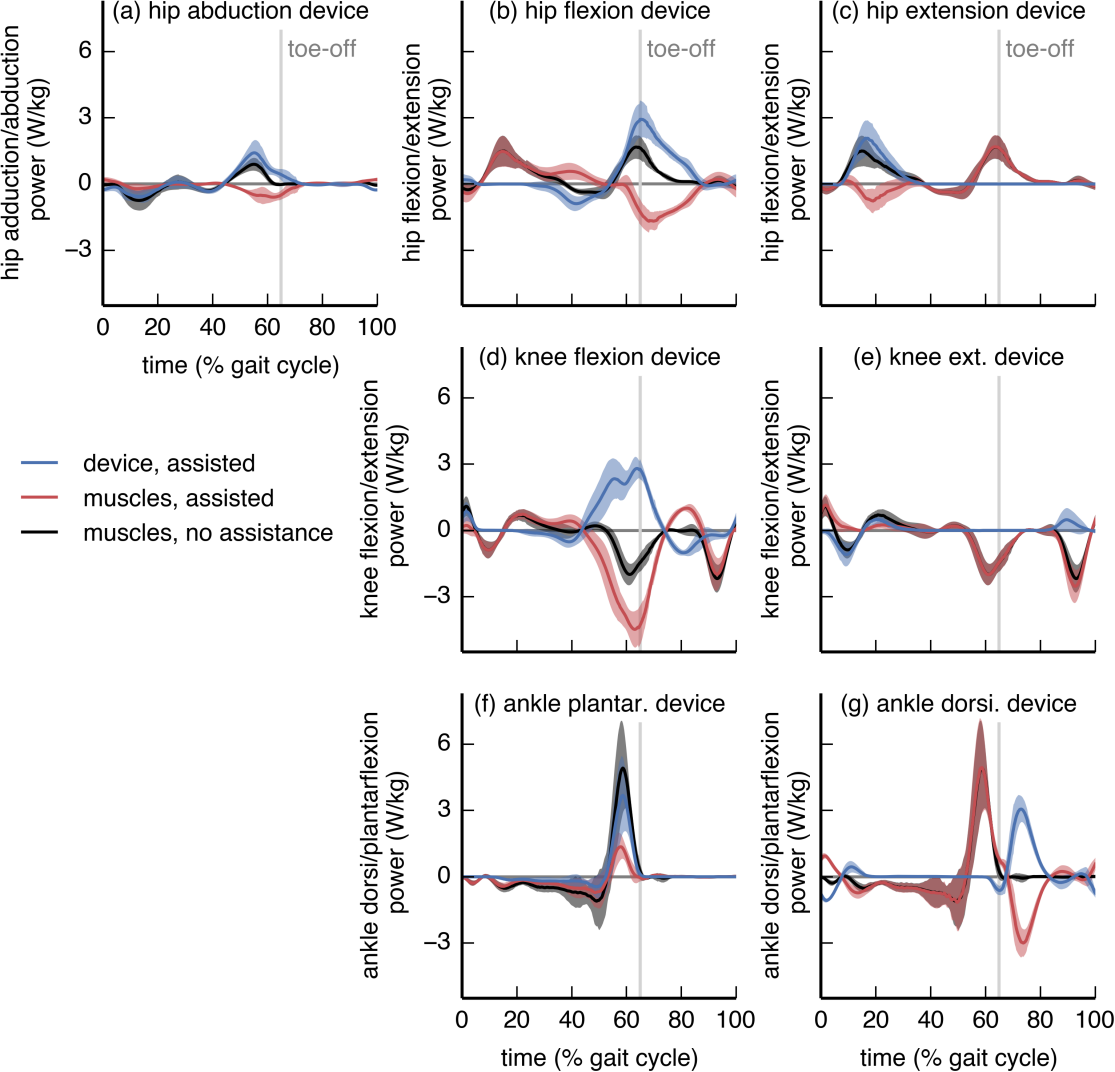
Device power profiles compared to net joint powers. The device actuator mechanical power (blue), net joint power generated by muscles in the presence of assistance (red), and net joint power generated by muscles when not being assisted (black) are shown for each device. All powers are normalized by subject mass. Curves are averages over 7 subjects; shaded regions indicate ±1 standard deviation. The “muscles, no assistance” power is the tracked net joint power for both the *no assistance* and *assisted* simulations; the “muscles, assisted” and “device, assisted” powers approximately sum to the “muscles, no assistance” power. Most devices exhibited substantially more positive work than negative work.

Most devices performed substantially more positive mechanical work than negative work (Fig 3 and Table 1). In general, the device power profiles resembled the corresponding net joint powers; notable exceptions were the ankle dorsiflexion and knee flexion devices. In some cases, instantaneous device power exceeded net joint power (hip abduction, hip flexion, knee flexion, and ankle dorsiflexion devices), suggesting that net joint power may not be a reliable guide for the power a device should inject.

### Muscle-generated joint moments

Each device changed the activity of many muscles. This occurred because the optimizer used the device to reduce overall muscle activation while seeking to achieve the same net joint moments as in the *no assistance* simulations. This effect of altered muscle activity is particularly clear when viewing muscle-generated joint moments, which we present for the ankle plantarflexion, knee flexion, hip flexion, and hip abduction devices.

**Fig 4:**
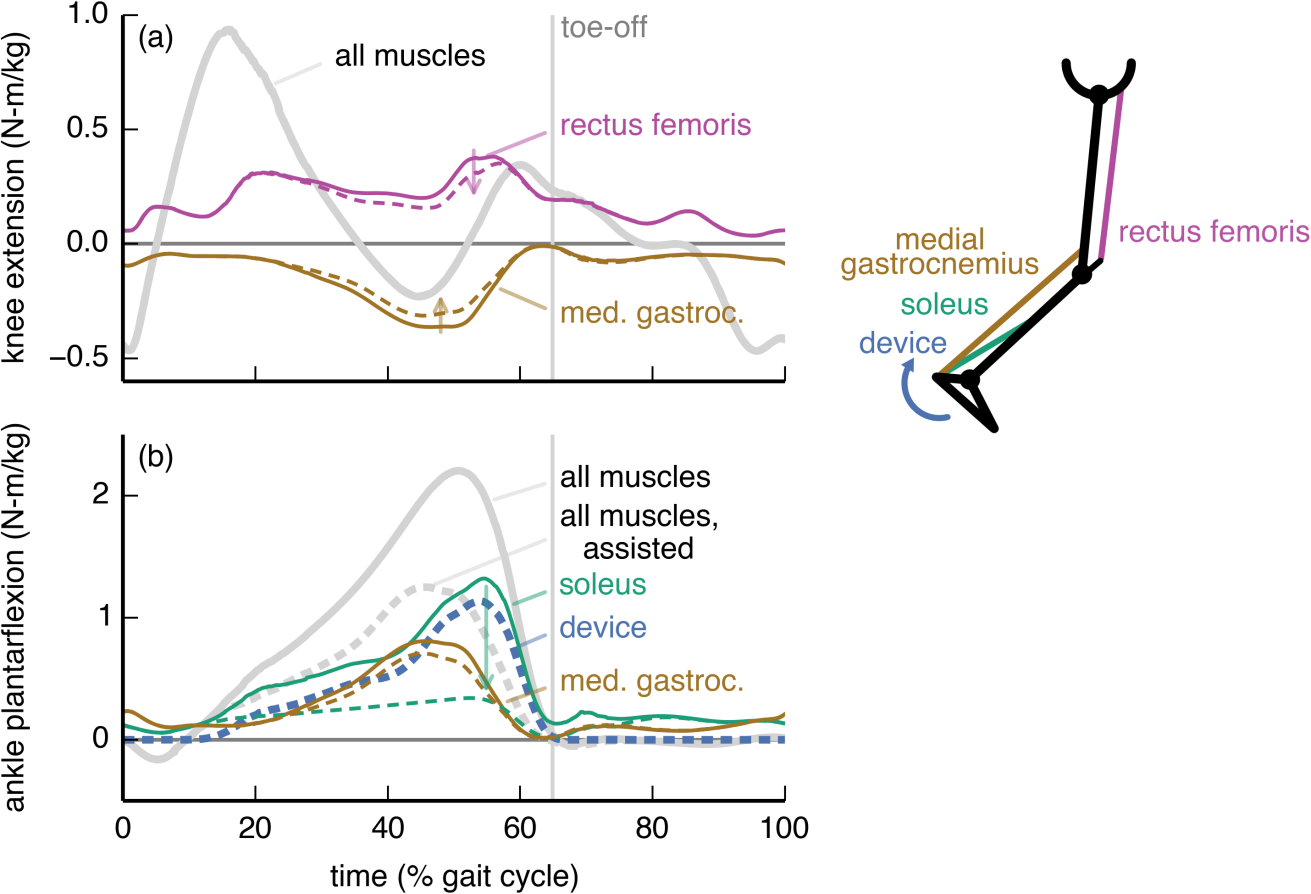
Ankle plantarflexion device: Joint moments from key muscles. The joint moments from the soleus (green), medial gastrocnemius (brown), and rectus femoris (purple) muscles about the (a) knee flexion/extension and (b) ankle dorsiflexion/plantarflexion degrees of freedom are shown without (solid) and with (dashed) the ankle plantarflexion device. For comparison, we also show the net moments from all muscles (gray) and from the device (dashed blue). The moments are normalized by subject mass and averaged across the 7 subjects. The ankle plantarflexion device largely bore the role of the soleus, while the gastrocnemius remained active to provide a knee flexion moment.

The ankle plantarflexion device affected predominantly the activity of the soleus muscle (Fig 4). The subject-average peak soleus ankle plantarflexion moment decreased by 74%. The medial gastrocnemius, however, remained active (12% decrease in peak ankle plantarflexion moment) to contribute to the required knee flexion moment. The device torque was thus less than the net ankle plantarflexion moment (solid gray curve in Fig 4B), and resembled the joint moment generated by the soleus when walking without assistance.

**Fig 5:**
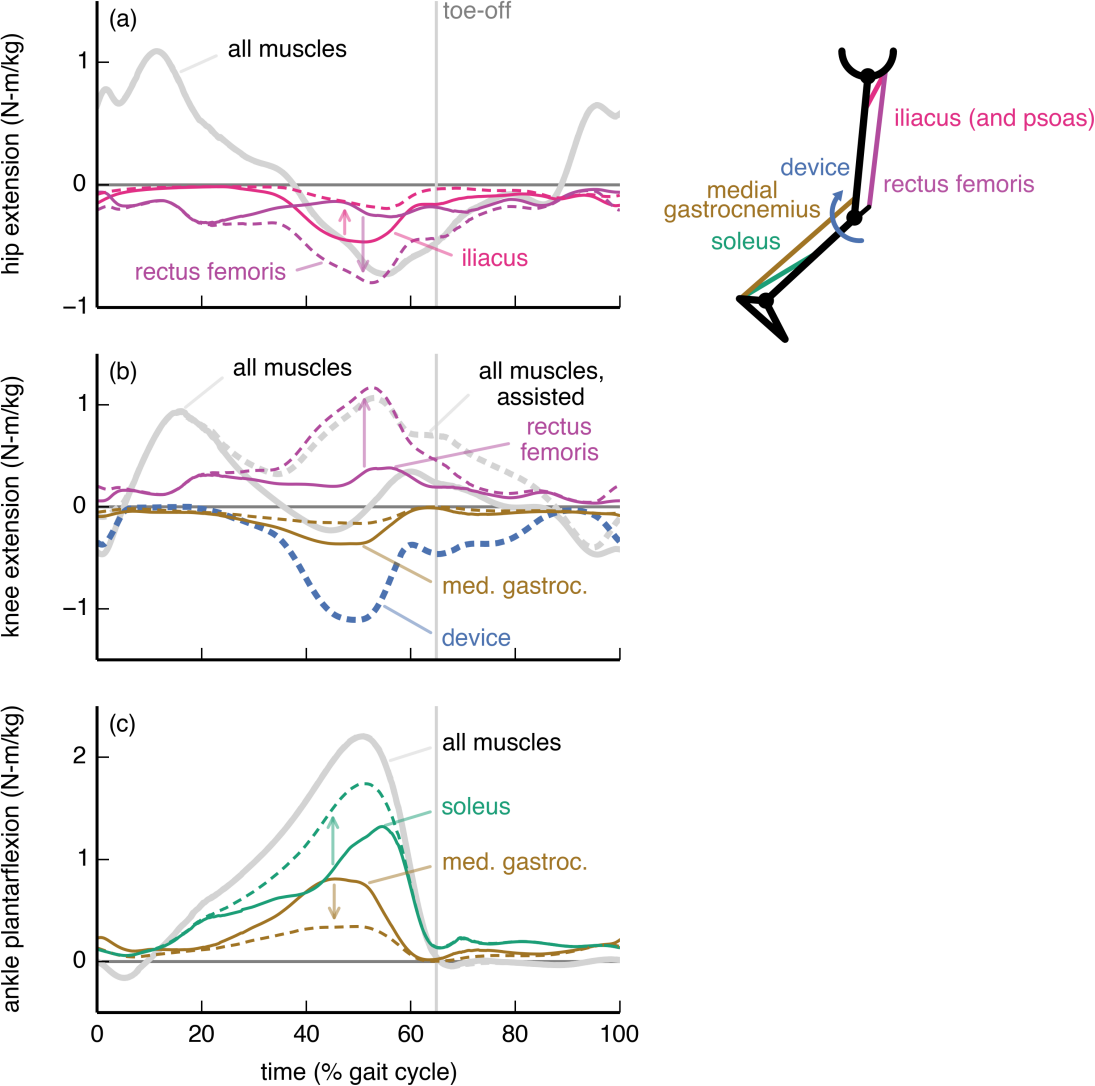
Knee flexion device: Joint moments from key muscles. The moments from the soleus (green), medial gastrocnemius (brown), rectus femoris (purple), and iliacus (pink) muscles about the (a) hip flexion/extension, (b) knee flexion/extension, and (c) ankle dorsiflexion/plantarflexion degrees of freedom. Muscle moments are shown without (solid) and with (dashed) the knee flexion device. For comparison, we also show the net moments from all muscles (gray) and from the device (dashed blue). The moments are normalized by subject mass and averaged across the 7 subjects. The device replaced much of the knee moment ordinarily generated by the gastrocnemius, which resulted in a decrease in gastrocnemius activity but an increase in the demand on the soleus to generate the plantarflexion moment. The rectus femoris countered the device to replace the iliacus and psoas (not shown) in providing a hip flexion moment.

The knee flexion device substantially affected the activity of the medial gastrocnemius, rectus femoris, soleus, and iliacus muscles (Fig 5). The device allowed the optimizer to decrease the activity of the medial gastrocnemius, a knee flexor (Fig 5B), though the muscle was still active to supply an ankle plantarflexion moment (Fig 5C). The activity of the soleus increased to compensate for the decrease in activity of the medial gastrocnemius. The rectus femoris applied a large knee extension moment that countered the device’s knee flexion moment; this occurred so that the rectus femoris could replace the iliopsoas (iliacus and psoas) in providing a hip flexion moment. This trade between the rectus femoris and iliopsoas was favorable in the optimization because the rectus femoris could produce a hip flexion moment more economically—less activation for the same joint moment—than could the iliopsoas.

**Fig 6:**
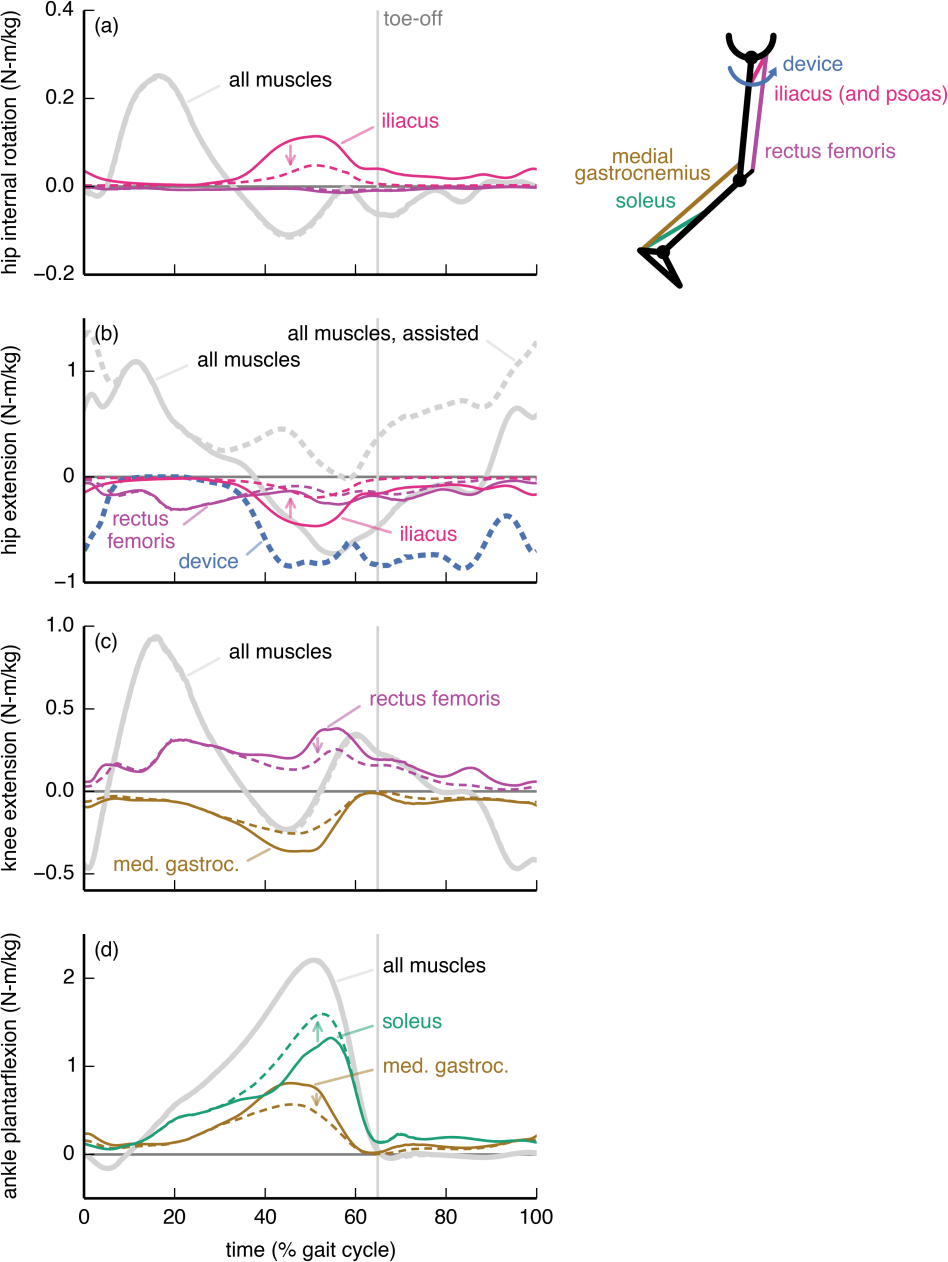
Hip flexion device: Joint moments from key muscles. The moments from the soleus (green), medial gastrocnemius (brown), rectus femoris (purple), and iliacus (pink) muscles about the (a) hip external/internal rotation, (b) hip flexion/extension, (c) knee flexion/extension, and (d) ankle dorsiflexion/plantarflexion degrees of freedom are shown without (solid) and with (dashed) the hip flexion device. For comparison, we also show the net moments from all muscles (gray) and from the device (dashed blue). The moments are normalized by subject mass and averaged across the 7 subjects. The device mostly replaced the iliacus—which normally counters the net hip internal rotation moment—and psoas (not shown). The device also partially replaced the rectus femoris, allowing for a decrease in co-contraction at the knee. The soleus replaced some of the ankle moment that had been provided by the gastrocnemius.

The hip flexion device affected muscles throughout the limb (Fig 6). The subject-average peak hip flexion moment from the iliacus, a large hip flexor, decreased by 58% with assistance (Fig 6B). This allowed a reduction in co-contraction for hip internal rotation, as the iliacus otherwise counters the net hip internal rotation moment in late stance. The device reduced the hip flexion moment from the rectus femoris, though to a smaller extent than for the iliacus. The rectus femoris also exerted a knee extension moment that countered the net knee flexion moment during late stance (Fig 6C). The reduction in rectus femoris activity allowed a reduction in co-contraction at the knee. This adaptation decreased the contribution from the medial gastrocnemius to the ankle moment, which the optimizer compensated for by increasing soleus activity.

**Fig 7:**
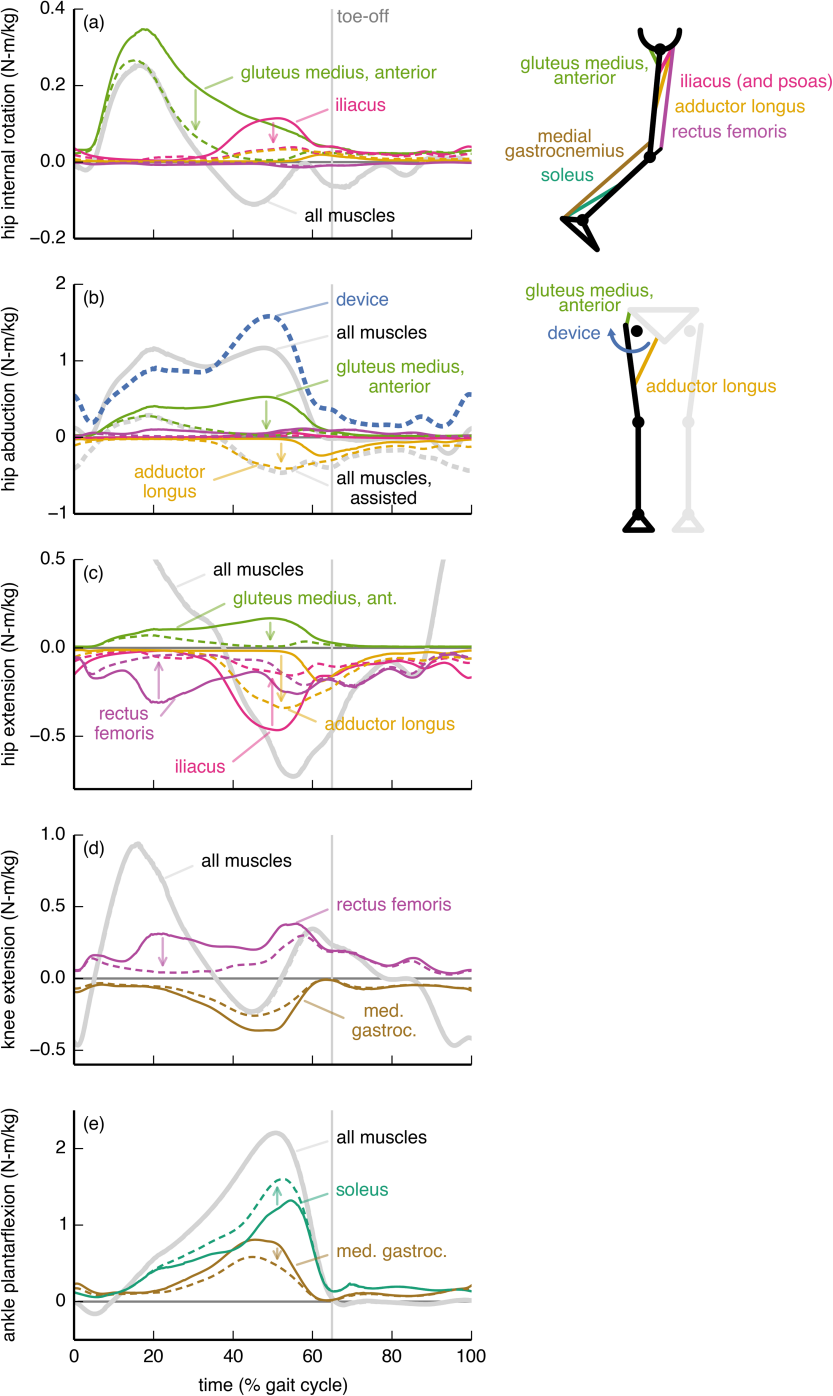
Hip abduction device: Joint moments from key muscles. The moments from the soleus (green), medial gastrocnemius (brown), rectus femoris (purple), iliacus (pink), adductor longus (orange), and gluteus medius (anterior portion; light green) muscles about the (a) hip external/internal rotation, (b) hip adduction/abduction, (c) hip flexion/extension, (d) knee flexion/extension, and (e) ankle dorsiflexion/plantarflexion degrees of freedom are shown without (solid) and with (dashed) the hip flexion device. For comparison, we also show the net moments from all muscles (gray) and from the device (dashed blue). The moments are normalized by subject mass and averaged across the 7 subjects. Without assistance, a large fraction of the hip abduction moment was generated by the gluteus medius. The device replaced the gluteus medius in late stance, but the gluteus medius remained active in early stance to provide a hip internal rotation moment. In late stance, the adductor longus countered the device to provide hip flexion more economically than could the iliacus or psoas (not shown).

The hip abduction device, like the hip flexion device, affected muscles throughout the limb (Fig 7). Without assistance, a large fraction of the hip abduction moment came from the anterior portion of the gluteus medius (Fig 7B). The device allowed the optimizer to reduce the gluteus medius hip abduction moment, though the reduction was larger in late stance than in early stance, as the muscle was still needed to generate a hip internal rotation moment in early stance (Fig 7A). In late stance, the adductor longus countered the device to provide a hip flexion moment (Fig 7C) more economically than could the iliopsoas. This adaptation allowed the iliacus to reduce its opposition to the net hip internal rotation moment (Fig 7A), resulting in an overall decrease in muscle activation despite the increase in adductor longus activity. To achieve the tracked net hip abduction moment in late stance, the device torque exceeded the net hip abduction moment. The hip abduction device allowed the adductor longus to replace some of the rectus femoris’ hip flexion moment, and thus also decreased co-contraction at the knee. As with the hip flexion device, this decrease in co-contraction at the knee resulted in an increase in soleus activity to achieve the necessary net ankle moment.

## Discussion

We simulated 7 hypothetical ideal devices and found that three of them yielded greater metabolic savings than our simulated ankle plantarflexion device (Fig 1). This is noteworthy given the current popularity of experimental ankle plantarflexion devices [9,13,14,22,50,56]. Because we directly estimated the metabolic savings achieved with different device locations, our study is an important step away from relying on indirect and coarse measures like positive joint power to decide where to assist. Part of the focus on ankle devices comes from the ankle’s large share of positive power output in walking [46,57,58]. Our results suggest that a device at a joint with high positive work, such as the ankle in loaded walking [46], does not necessarily yield the highest metabolic savings [19]. Nevertheless, all the devices we explored warrant consideration from device designers: reducing metabolic rate by even 5%—as is possible with a hip extension device [59]—is tantamount to removing 4 kg from a torso load [45] and would markedly help load carriers.

### Hip abduction device

The metabolic savings from the hip abduction device were surprisingly large, given that walking is a predominantly sagittal-plane motion. Hip abduction has a low net joint power (Fig 3), so it is unlikely that a joint-level power analysis would produce interest in assisting hip abduction. However, muscles consume energy even when not performing work (e.g., when contracting isometrically), and hip abduction has a substantial metabolic rate during loaded walking (S4 Fig).

The hip abduction device is even more attractive when considering its relatively low power requirements: this device had the greatest ratio of metabolic savings to peak instantaneous positive device power and the greatest ratio of metabolic savings to *average* positive device power (Table 1). These metrics can be used to estimate, respectively, the increase in metabolic cost from carrying the mass of a device and the duration for which an untethered device could operate (that is, battery life); see the “Evaluating the devices” section, above, for an explanation. Notably, the hip abduction device performed similar amounts of positive and negative mechanical work (the ratio of average positive to average negative power was 1.6; Table 1), suggesting that a hip abduction device could incorporate passive components to reduce its power consumption. Thus, a hip abduction device could weigh less and operate for longer than devices placed elsewhere, especially since its mass could be located more proximally than that of devices that assist the ankle or knee [49]. Given these benefits, we suggest that experimentalists further pursue the feasibility and performance of hip abduction devices for reducing metabolic cost.

### Optimal device torques and the underlying musculature

To maximize metabolic savings, device torque profiles should generally differ from the net joint moments; this is because human joints are driven by muscles and not simple torque actuators. In particular, the optimal device torque profiles and the corresponding changes in muscle activity we observed were shaped by the presence of muscles that actuate multiple degrees of freedom (this includes biarticular muscles as well as muscles crossing a single joint that has multiple degrees of freedom). We found that a device torque may be less than the corresponding net joint moment if an assisted muscle also contributes to the required net joint moment about another joint motion (e.g., ankle plantarflexion device and medial gastrocnemius [60,61]; Fig 4A). Conversely, if a muscle crossing an assisted joint motion generates an undesired moment about another joint motion, then the device can largely take over for this muscle (e.g., hip flexion device and iliacus; Fig 6A). Surprisingly, device torques may also exceed net joint moments to allow antagonistic muscles to take over for less economical muscles (in terms of joint moment per unit activation) at other degrees of freedom (e.g., knee flexion device and rectus femoris; Fig 5B). Designers can use our optimal device torque profiles as guidelines for choosing the timing and magnitude of assistive torques that take into account musculotendon dynamics, musculoskeletal geometry, and muscle energetics. Additionally, simulated optimal torque profiles can narrow the range of experimental conditions required to find a device’s optimal performance [62]. Our results may also help understand how and why muscle activation patterns change in response to applied torques.

Devices can affect the activity of muscles that do not span the assisted degree of freedom—for example, the knee flexion, hip flexion, and hip abduction devices all affected soleus activity. There is experimental support for this observation: Lenzi et al. [63] created a hip flexion/extension device that reduced medial gastrocnemius activity in walking. To exploit coupling across degrees of freedom, we suggest that experimentalists devote more attention to devices that actuate multiple degrees of freedom [17,18]. For example, unlike the strictly ankle plantarflexion device we presented (Fig 4), a device that provides both ankle plantarflexion and knee flexion moments may decrease activity of the gastrocnemius substantially. Indeed, Quinlivan et al. [18] have shown large metabolic savings with a device that simultaneously assists ankle plantarflexion and hip flexion.

### Study limitations

This study has a number of limitations that require consideration when interpreting our results. We assumed that subjects walked with the same kinematics (and ground reaction forces) when assisted. Some experimental exoskeleton studies report relatively small changes in kinematics or joint moments with assistance [9,17,20,25,64,65], while others report much larger changes [12,18,61,63]. Our simulations also do not capture the effects of training protocols or long-term adaptation to the devices, which are important considerations during experimental testing of devices [20,21].

We were interested in devices that minimize metabolic cost, yet we minimized the sum of squared muscle activations. We chose this objective function because minimizing activations is more computationally tractable, activation is a dominant variable in the metabolics model [35], and muscle activity correlates well with metabolic cost [25,32,66]. Simulations that allow kinematics to adapt and that minimize metabolic cost directly may reveal assistance strategies that are even more metabolically beneficial than those we presented, and could alter the ranking of the devices’ metabolic savings. On the other hand, it may be desirable in some situations to retain normal kinematics, as altered kinematics could have negative side effects (e.g., increased joint loading).

Our simulations produced muscle activity in the medial hamstrings, biceps femoris short head, and gastrocnemius (early swing) that was not present in our electromyography measurements (S2 Fig). This excess activity resulted from excessive passive force generated by the knee extensor muscles, a known problem with some musculoskeletal models [47]. We found that removing the knee extensor passive force in the model reduced the metabolic savings of the hip flexion device (from 18 to 15%) and knee flexion device (from 13 to 8%). Removing passive knee extensor force did not substantially affect the metabolic savings of the hip abduction or ankle plantarflexion devices, did not change the overall nature of the muscle adaptations, and does not affect the main conclusions of our study. Our simulations also produced excessive tibialis anterior activity in swing; as a result, the reported metabolic savings for the ankle dorsiflexion device are likely inflated.

Several factors influence the uncertainty in our predictions for changes in metabolic cost. Our simulations estimated a 40% increase in cost with load while indirect calorimetry during our experiments estimated a 48% increase, suggesting that our simulations also underestimated reductions in cost with assistance. Another potential source of underestimation in metabolic rate is our constraint that kinematics could not change between the *no assistance* and *assisted* simulations. Still, our predicted metabolic savings seem reasonable in comparison to published experimental studies. Our ideal massless ankle plantarflexion device resulted in an 8.7% reduction in *gross* metabolic rate (approx. 11% in *net* metabolic rate), which is greater than the 8.0% reduction in *net* metabolic rate reported when using an ankle plantarflexion device during a loaded walking experiment [14]. Ding et al. [59] achieved a 5.7–8.5% reduction in net metabolic rate (compared to wearing the device unpowered) with a hip extension device for loaded walking, which is similar to our reduction of 4.9% in gross metabolic rate. However, our simulations produced device torques and changes in muscle activity that were much greater than what experimentalists have observed [9,12,16,17,59], so it is expected that our predicted reductions should exceed experimental reductions.

Considering these limitations, the value of this study is in the ranking of the metabolic savings and power requirements of the devices, and the qualitative insights we obtained about how muscle activity may change with assistance. Without accounting for kinematic adaptation, neural constraints, training protocols, and other practical matters, it is unreasonable to expect a close quantitative match in metabolic reductions, device torques, and muscle activity adaptations between experiments and our simulations.

### Summary of insights

Our experience using muscle-driven simulations to study uniarticular assistance strategies have led to the following qualitative insights:

1. Most experimentalists have focused on assisting the ankle, yet assisting the hip or knee has the potential to lead to greater metabolic savings than assisting the ankle.
2. Assisting hip abduction may be an effective strategy to reduce metabolic cost, yet this strategy is largely unexplored.
3. Devices that assist one joint can affect the activity of muscles that do not span that joint.
4. The activity of an assisted muscle may remain if the muscle provides a beneficial action at an unassisted degree of freedom.
5. Joint-level moment and power analyses may not sufficiently explain the relative performance of a device because optimal device torques sometimes differ from the net joint moments and device performance is sensitive to details of the device torque profiles.

### Future work

In light of the study limitations, future studies should employ simulation approaches that examine how changes in kinematics may affect the performance of a device (“predictive simulation”) [29,67]. To obtain more realistic results, predictive simulations could model non-ideal aspects such as device mass and actuator torque and power limits. To make stronger conclusions from simulations (e.g., discovering the maximum achievable savings), studies should be performed with larger sample sizes. Simulation experts and experimentalists should work together to improve the accuracy of simulated exoskeletal assistance through comparison of simulated muscle activations with experimental recordings of muscle activity and further testing of changes in metabolic cost with load and assistance. Device designers must tackle issues we could not; for example, a hip abduction device will require effective means of transmitting forces to the skeleton.

Future studies should look beyond metabolic cost, as optimizing a device to minimize solely metabolic cost may worsen muscle fatigue, joint loading, and joint stability [10]. For example, our knee flexion device caused very high rectus femoris activation during lengthening, which is likely to cause fatigue [68]. Our hip flexion device decreased co-contraction at the knee, which could decrease knee stability [69]. Furthermore, devices that explicitly optimize the ratio of metabolic savings to device power may achieve greater values for this ratio than did our hip abduction device. Novel methods that allow flexible objective functions could discover such devices, and could also optimize other metrics that would improve safety, comfort, and performance.

## Conclusions

In this study, we used musculoskeletal simulation to evaluate how 7 hypothetical, ideal, bilateral assistive devices affected muscle activity and metabolic cost when walking with heavy loads. This work provides a foundation for understanding the musculoskeletal factors that may affect device performance. We also provided suggestions to device designers, which can serve as a springboard for deciding which devices to create next. In particular, we are excited for designers to create hip abduction devices that incorporate passive components, and to explore devices that actuate multiple degrees of freedom.

The insights we gained in this study relied on the use of musculoskeletal and metabolics models. These models can reveal insights that are difficult to discover via experiments alone; for example, we found that devices may substantially affect the metabolic rate of joint motions other than the one being assisted. Our findings support use of musculoskeletal modeling and simulation to predict how hypothetical devices may perform and to understand the performance of actual devices [22,28,60]. This work complements experiments, which are necessary to test the accuracy of the predictions made by simulations, improve musculoskeletal and metabolics models, and solve the practical challenges we ignored. We invite other researchers to use our data and code (freely available at https://simtk.org/home/assistloadwalk) to build upon our work.

## Supporting information

S1 Table Demographics of subjects. (PDF)

S1 Fig Joint angles and net joint moments for the *no load* and *loaded* conditions. Joint angles (left) and net joint moments from muscles (normalized by subject mass; right) are shown for the simulations of the *no load* (green) and *loaded* (black) conditions for the hip adduction/abduction (top), hip flexion/extension, knee flexion/extension, and ankle dorsiflexion/plantarflexion (bottom) degrees of freedom. Curves are averages over 7 subjects; shaded regions indicate ±1 standard deviation. The vertical lines indicate average toe-off time for the two conditions.
(TIF)

S2 Fig Simulated and measured muscle activity for the *no load* and *loaded* conditions. Each graph compares the electromyography measurements (black) of the muscle listed on the left to the simulated activation (unitless, between 0 and 1; blue, green, red) of the relevant muscles in the model. Electromyography measurements were first band-pass filtered (50–500 Hz), then rectified, and finally low-pass filtered (7.5 Hz). We normalized the electromyography data by the maximum value observed across all four experimental conditions for a given subject and sensor. Curves are averages over 7 subjects; shaded regions indicate ±1 standard deviation. Electromyography data were collected on the right leg, but activation is averaged over both the left and right legs of each subject. (*posterior*, *intermed*., and *anterior* correspond to muscle–tendon units 3, 2 and 1, respectively, in the model; *semimem*.: semimembranosus; *semiten*.: semitendinosus).
(TIF)

S3 Fig Validation of metabolics estimates. Graph (a) shows gross average whole-body metabolic rate normalized by subject mass and walking speed (W/kg/(m/s)) for the *no load* and *loaded* conditions, obtained with indirect calorimetry (triangles, dashed lines) and with the simulations (circles, solid lines). Each triangle comes from the last minute of 7 minutes of treadmill walking for a single subject and condition. Each circle is obtained by averaging across 3 trials for a single subject and condition; error bars provide the standard deviation across these 3 trials. Each color represents a single subject. Graph (b) shows the same data as (a) but displayed as simulation versus indirect calorimetry, with a linear regression fit (dashed gray) and a *y = x* line (solid gray). We refer to the simulations of the *loaded* condition as *no assistance*, as they are the baseline for the simulations of assistance. The simulations appear to underestimate the change in metabolic rate between conditions.
(TIF)

S4 Fig Metabolic rate attributed to joint motions, without and with assistance, for each device. Most devices only partially reduced the metabolic rate of its associated joint motion. Each graph shows the metabolic rate (horizontal axis) for a single device that we attributed to 8 *joint motions* (one direction of a degree of freedom; vertical axis), summed over the same joint motion for both legs and averaged over the gait cycle, without (white) and with (gray) assistance. The metabolic rate we attributed to a joint motion comes from all the muscles that actuate the joint motion, apportioned according to the muscles’ moment arms; see Eq (6). Dots to the left of the bars denote the joint motion being assisted by the device. Not all joint motions are shown (namely, hip rotation). The length of each bar indicates an average over 7 subjects; whiskers indicate ±1 standard deviation.
(TIF)
