# Supplementary material for: Simulating ideal assistive devices to reduce the metabolic cost of walking with heavy loads
Source: PLoS One. 2017 Jul 12;12(7):e0180320. doi: 10.1371/journal.pone.0180320 (PMC5507502; doi:10.1371/journal.pone.0180320)
Supplement: S1 Table — (PDF) [file pone.0180320.s001.pdf]

Subject	Sex	Age (years)	Height (m)	Mass (kg)
1	M	27	1.92	112
2	M	20	1.88	89
3	M	19	1.91	87
4	M	21	1.80	64
5	M	31	1.83	85
6	M	32	1.83	67
7	M	27	1.83	84
