# Supplementary figures and images for: Simulating ideal assistive devices to reduce the metabolic cost of walking with heavy loads
Source: PLoS One. 2017 Jul 12;12(7):e0180320. doi: 10.1371/journal.pone.0180320 (PMC5507502; doi:10.1371/journal.pone.0180320)

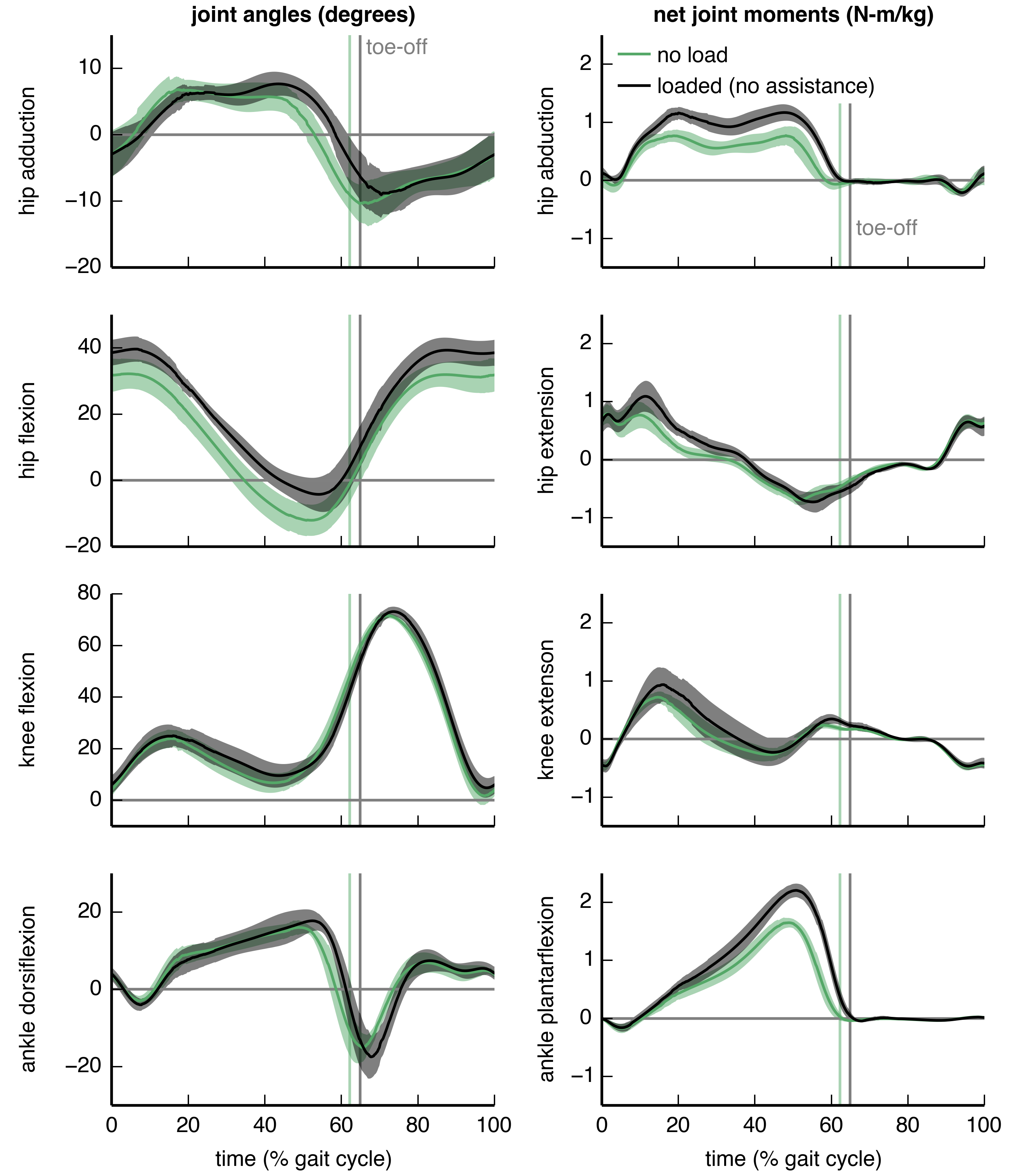

Supplement: S1 Fig — Joint angles (left) and net joint moments from muscles (normalized by subject mass; right) are shown for the simulations of the no load (green) and loaded (black) conditions for the hip adduction/abduction (top), hip flexion/extension, knee flexion/extension, and ankle dorsiflexion/plantarflexion (bottom) degrees of freedom. Curves are averages over 7 subjects; shaded regions indicate ±1 standard deviation. The vertical lines indicate average toe-off time for the two conditions. (TIF) [file pone.0180320.s002.tif]

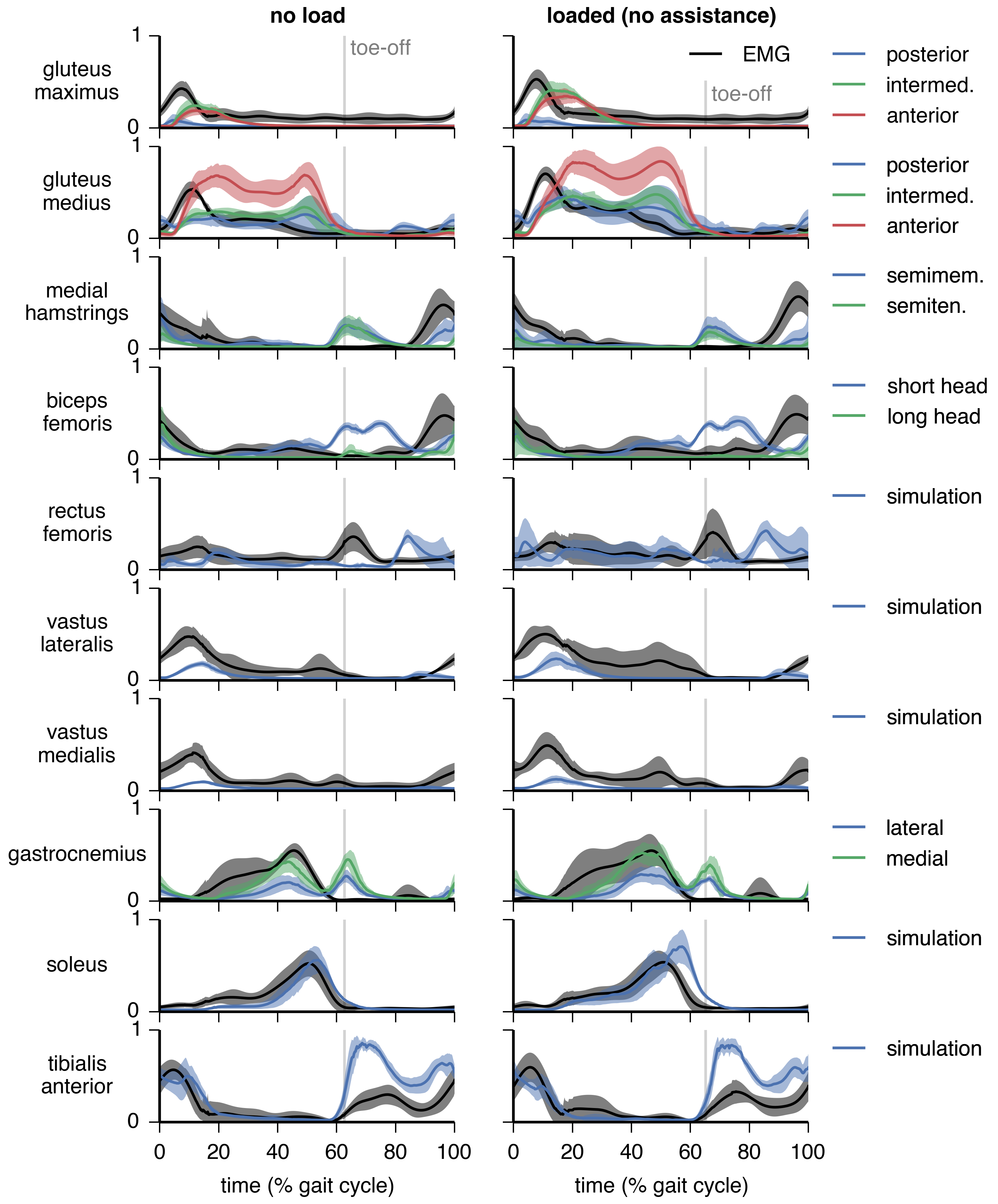

Supplement: S2 Fig — Each graph compares the electromyography measurements (black) of the muscle listed on the left to the simulated activation (unitless, between 0 and 1; blue, green, red) of the relevant muscles in the model. Electromyography measurements were first band-pass filtered (50–500 Hz), then rectified, and finally low-pass filtered (7.5 Hz). We normalized the electromyography data by the maximum value observed across all four experimental conditions for a given subject and sensor. Curves are averages over 7 subjects; shaded regions indicate ±1 standard deviation. Electromyography data were collected on the right leg, but activation is averaged over both the left and right legs of each subject. (posterior, intermed., and anterior correspond to muscle–tendon units 3, 2 and 1, respectively, in the model; semimem.: semimembranosus; semiten.: semitendinosus). (TIF) [file pone.0180320.s003.tif]

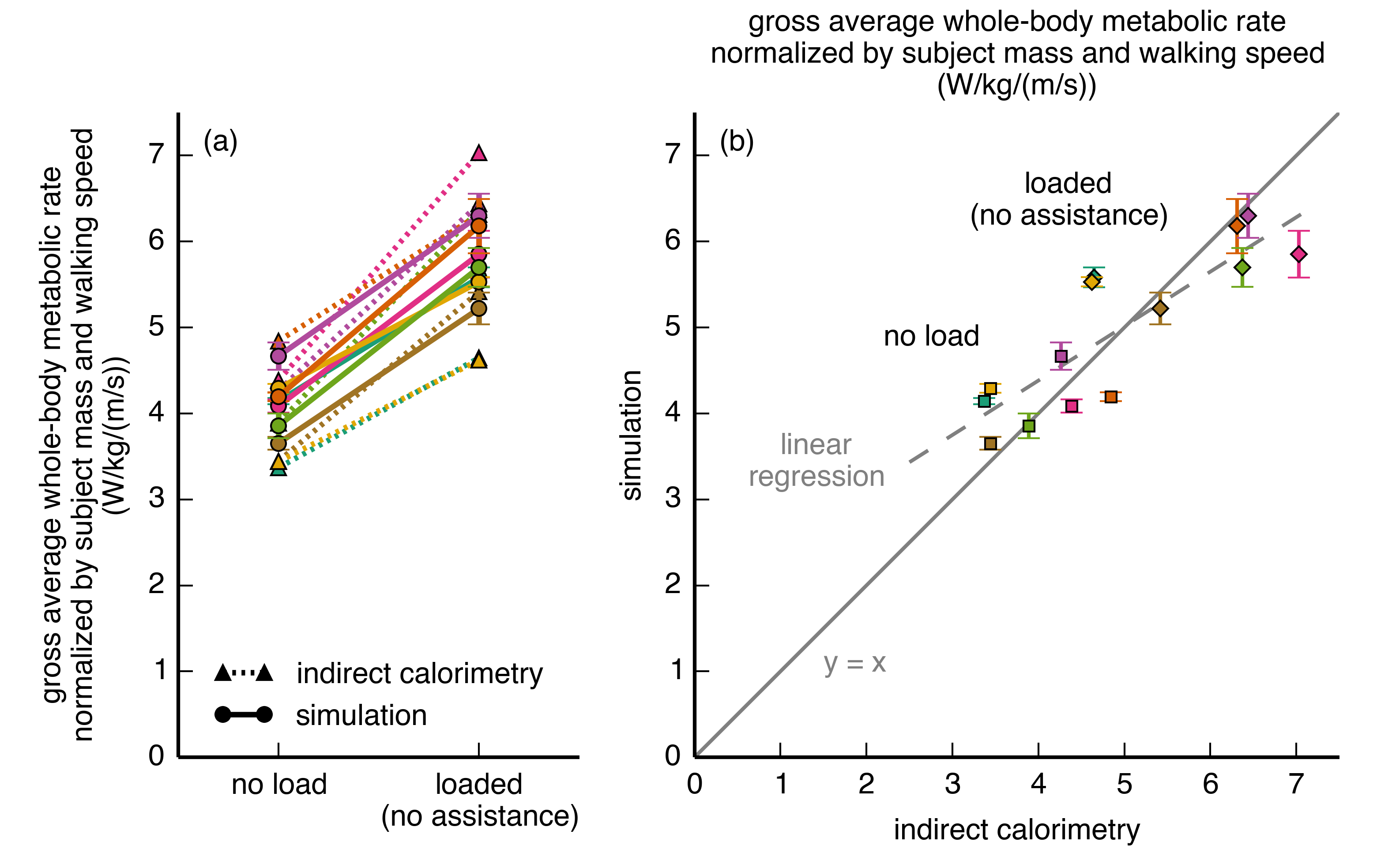

Supplement: S3 Fig — Graph (a) shows gross average whole-body metabolic rate normalized by subject mass and walking speed (W/kg/(m/s)) for the no load and loaded conditions, obtained with indirect calorimetry (triangles, dashed lines) and with the simulations (circles, solid lines). Each triangle comes from the last minute of 7 minutes of treadmill walking for a single subject and condition. Each circle is obtained by averaging across 3 trials for a single subject and condition; error bars provide the standard deviation across these 3 trials. Each color represents a single subject. Graph (b) shows the same data as (a) but displayed as simulation versus indirect calorimetry, with a linear regression fit (dashed gray) and a y = x line (solid gray). We refer to the simulations of the loaded condition as no assistance, as they are the baseline for the simulations of assistance. The simulations appear to underestimate the change in metabolic rate between conditions. (TIF) [file pone.0180320.s004.tif]

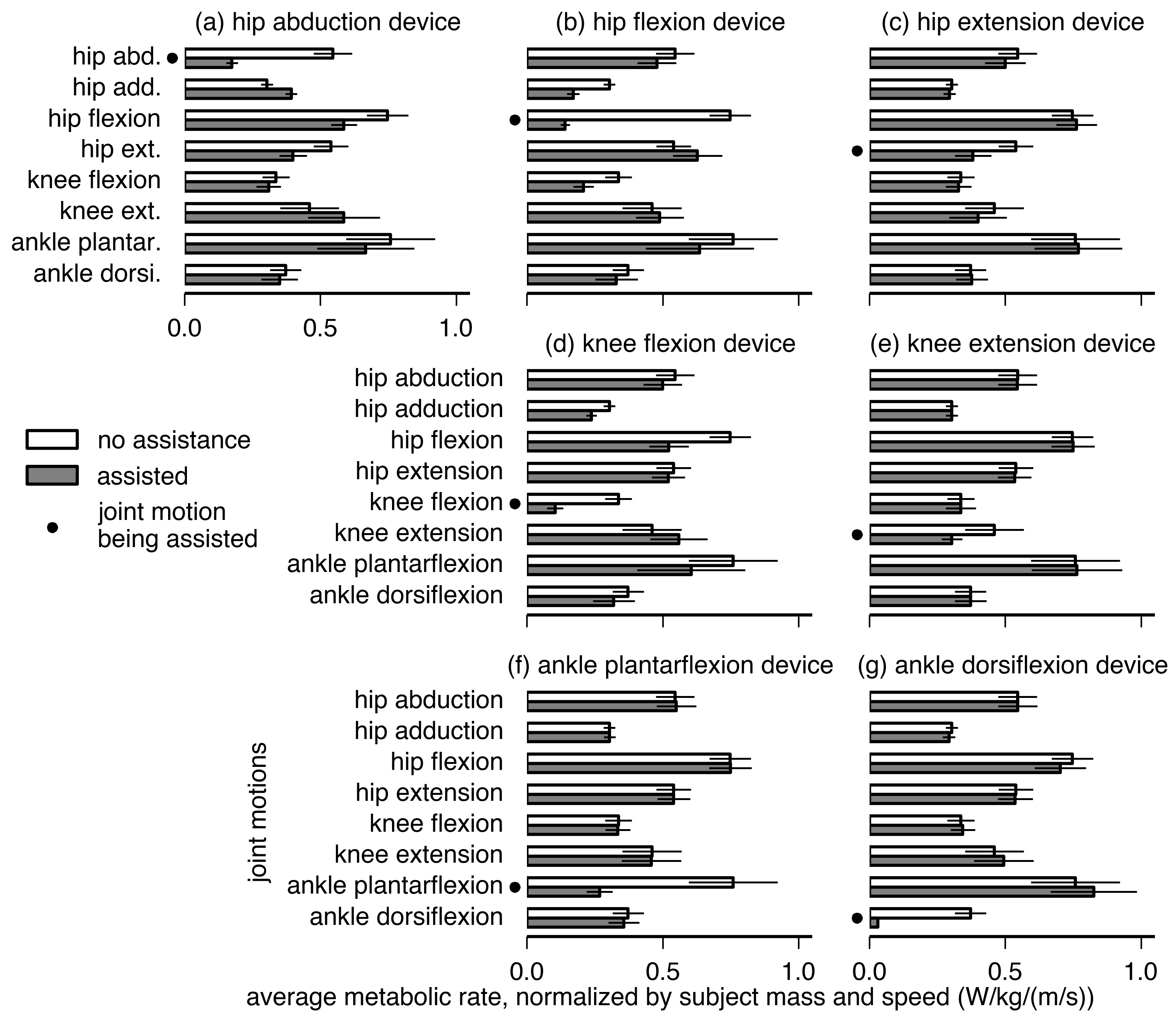

Supplement: S4 Fig — Most devices only partially reduced the metabolic rate of its associated joint motion. Each graph shows the metabolic rate (horizontal axis) for a single device that we attributed to 8 joint motions (one direction of a degree of freedom; vertical axis), summed over the same joint motion for both legs and averaged over the gait cycle, without (white) and with (gray) assistance. The metabolic rate we attributed to a joint motion comes from all the muscles that actuate the joint motion, apportioned according to the muscles’ moment arms; see Eq (6). Dots to the left of the bars denote the joint motion being assisted by the device. Not all joint motions are shown (namely, hip rotation). The length of each bar indicates an average over 7 subjects; whiskers indicate ±1 standard deviation. (TIF) [file pone.0180320.s005.tif]
